# Long-term decitabine/retinoic acid maintenance treatment in an elderly sAML patient with high-risk genetics

**DOI:** 10.1186/s13148-023-01596-5

**Published:** 2023-11-28

**Authors:** Nora Rebeka Javorniczky, Olga Grishina, Inga Hund, Milena Pantic, Dietmar Pfeifer, Claudia Schmoor, Johanna Thomas, Justus Duyster, Heiko Becker, Michael Lübbert

**Affiliations:** 1 Department of Hematology, Oncology and Stem Cell Transplantation, Faculty of Medicine, University Medical Center Freiburg, University of Freiburg, Hugstetterstr. 55., 79106 Freiburg, Germany; 2 Clinical Trials Unit, University Medical Center Freiburg, Faculty of Medicine, University of Freiburg, Freiburg, Germany; 3 German Cancer Consortium (DKTK) and German Cancer Research Center (DKFZ), Partner Site Freiburg, Freiburg, Germany

## Abstract

Elderly patients with AML ineligible for induction have a dismal prognosis; hence disease stabilization is a primary treatment goal. This case of a 75-year-old patient with secondary AML receiving the combination of decitabine and ATRA (within the DECIDER trial, NCT00867672) demonstrates an above-average survival. The therapy administered over 52 cycles led to complete molecular and hematological remission and resulted in 5.3 years overall survival. Clonal evolution of the leukemic clone could be demonstrated using DNA sequencing methods. According to the literature, this case constitutes the longest continued HMA exposure in an elderly AML patient ineligible for standard chemotherapy.

**Supplementary Information:**

The online version contains supplementary material available at 10.1186/s13148-023-01596-5.

## Introduction

DNA-hypomethylating agents (HMA) are increasingly administered over a prolonged period of time, with the aim of maintaining an objective hematologic response or at least disease stability, in patients with acute myeloid leukemia (AML) and myelodysplastic syndrome (MDS). Hence, in many AML/MDS patients ineligible for intensive chemotherapy and allografting, continued HMA-based treatment has become the mainstay of therapy, with the goal of stabilizing quality of life and limiting inpatient hospital stays. However, achieving administration of more than 20 treatment cycles is uncommon, since even when HMAs are combined with venetoclax (VEN), secondary resistance will eventually develop [1].

## Methods

The index patient was included in the DECIDER study in April 2014. DECIDER is a prospective, randomized, observer blind, parallel group, multicenter, Phase II study with a 2 × 2 factorial design. The study cohort included AML patients aged 60 years or older and unfit for standard induction chemotherapy. Patients were randomized to one of the four treatment groups: decitabine (DAC) alone or in combination with valproic acid (VPA) or all-*trans* retinoic acid (ATRA) or with both add-on drugs. During the study treatment, the patient attended medical examination on a monthly basis. For the initial diagnosis and the follow-ups the following diagnostic examinations were performed: peripheral blood and bone marrow (BM) cytomorphology, immunophenotyping and 54-gene myeloid panel sequencing (Additional file 2). Whole-exome sequencing (WES) was performed at the time of diagnosis and shortly before the patient's death.

## Results

Here, we report on a 74-year-old female patient diagnosed in 2013 with multilineage MDS with excess of BM blasts (< 10%), deletion of the long arm of chromosome 5 (del(5q)) and red blood cell transfusion requirements. Therapy with lenalidomide was initiated, with subsequent blood count and fluorescence in situ hybridization (FISH) studies demonstrating a very good response.

**Fig. 1:**
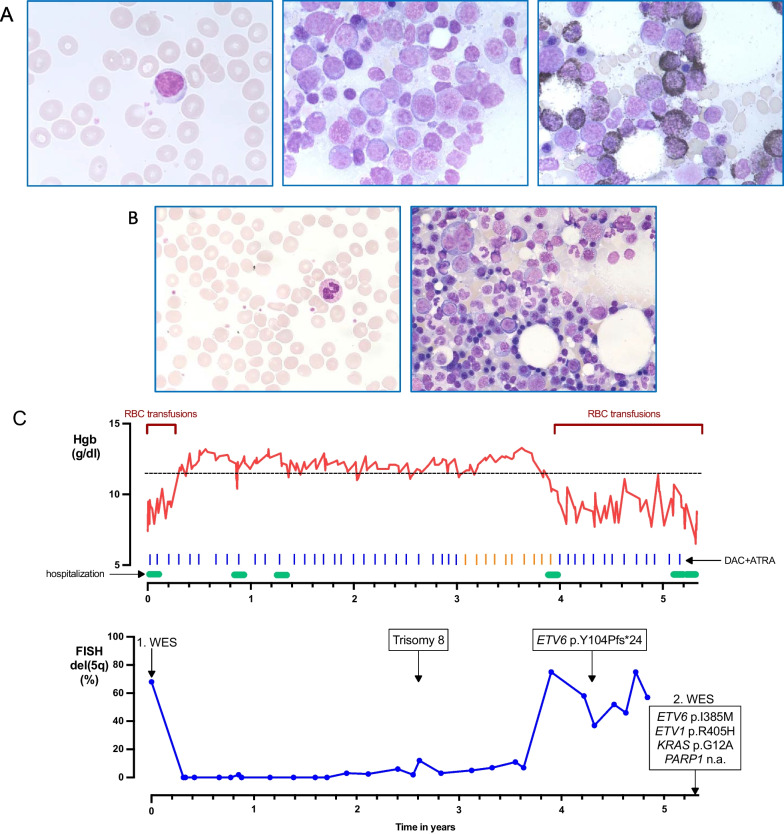
**A** Cytological diagnosis of tAML. Left panel myeloblast in PB smear, middle panel population of blasts (> 50%) in BM (Pappenheim-staining), right panel POX-positive blasts in BM (POX-staining), original magnification × 63. **B** Best response to DAC + ATRA. Left panel PB smear with mature neutrophil granulocyte, right panel BM with normal trilineage hematopoiesis (Pappenheim-staining), original magnification × 40. **C** Kinetics of Hgb level (upper graph, red line), RBC transfusion requirements and timepoints of inpatient hospitalization. Bars depicting each DAC cycle (blue d1-5, orange d1-3). Lower graph showing proportion of del(5q) (blue line) detected with FISH, emerging mutations are depicted at the timeline. Timeframe over 5 years from therapy initiation with DAC + ATRA until patient deceased. *Hgb* Hemoglobin, *PB* Peripheral blood, *POX* Peroxidase, *RBC* Red blood cell

Fourteen months after the initial diagnosis of MDS, progressive pancytopenia with 4% blasts in the peripheral blood developed. BM cytomorphology revealed transformation in AML with 56% blasts. (Table 1; Fig. 1A) FISH analysis confirmed the known del(5q) in 90% of BM cells; no mutations were detected with the 54-gene myeloid panel sequencing (NGS). As the patient was ineligible for conventional induction chemotherapy, she was enrolled on the randomized phase II DECIDER study (NCT00867672), testing DAC alone, in combination with VPA, ATRA or with both drugs [2]. She was randomized in arm C (DAC 20 mg/m^2^ d1-d5, ATRA 45 mg/m^2^ d6-28). After 2 therapy cycles the BM blasts dropped to 16%, after 3 cycles she became transfusion-independent, after 6 cycles a complete hematological and molecular remission (FISH: del(5q) [0/200]) was achieved. (Fig. 1B) Therapy was administered every 4 weeks for 29 cycles, with further lasting complete hematological remission. (Table 1) From cycle 30, a reduction of the DAC administration from 5 to 3 days was performed.

**Table 1:** Laboratory data and FISH at initial diagnosis of AML and at time of best response (1.4 years after therapy initiation)

Variable	Reference range	Timepoints of laboratory diagnostics	
		Initial diagnosis of AML	Best response
White blood cells, × 10^9^/L	4.0–10.4	1.38	4.24
Absolute neutrophil count, × 10^9^/L	1.9–7.3	0.04	1.15
Platelets, × 10^9^/L	176–391	62	188
RBC, × 10^12^/L	4.0–5.2	2.25	3.57
Hgb, g/dl	11.6–15.5	7.4	11.9
Blasts in BM, %	0.3–5.0	56	2
Blasts in PB, %	0	4	0
FISH: del(5q31/5q33) in PB, %	0	68	0

*Hgb* Hemoglobin, *PB* Peripheral blood, *RBC* Red blood cell

After the 40th treatment cycle, the hemoglobin level decreased, and the BM cytomorphology study demonstrated a blast count of 10%. The dose of DAC was again elevated to 5 days per cycle, which resulted in increasing hemoglobin levels. After 43 treatment cycles (4.2 years from AML diagnosis), NGS of peripheral blood revealed a new mutation in *ETV6* exon 3: p.Y104Pfs*24, with 12% variant allele frequency (VAF).

After 52 cycles of DAC + ATRA (5.3 years from AML diagnosis), marked pancytopenia with an increasing peripheral blast count up to 34% was observed, further specific treatment was withheld, and shortly thereafter the patient died of pneumonia and acute respiratory distress syndrome.

## Discussion

The DECIDER trial was designed to address whether the add-on of VPA or ATRA would increase the overall response rate (ORR), overall (OS), and event-free survival (EFS). The results demonstrated a higher ORR and prolonged OS with ATRA but not VPA [2]. Prompted by the case described here, we analyzed all patients treated on the DECIDER trial who lived for more than 5 years from start of treatment. Of a total of 6 patients (last follow-up 04/2021), only one other patient had received continuous study treatment (39 cycles of DAC + VPA), the other patients had received an allogeneic stem cell transplantation (*n* = 3), or 7 + 3 salvage for early progressive disease (*n* = 1, ELN favorable risk). Conducting a PubMed-based literature search for the maximum number of HMA-cycles administered and reported in large phase II/III AML trials and a case series of HMA long-term responders, we could not identify a patient having received more than 49 treatment cycles. [1, 3–7] (Additional file 1: Table S1) These results confirm that the presented case with 52 cycles is indeed exceptional.

The continuous application of ATRA over 5 years, at the daily dose administered intermittently in acute promyelocytic leukemia (APL), led to a much greater cumulative drug exposure than that administered in APL. Notably, we observed a very good drug tolerability without ATRA-related toxicities. The DECIDER study demonstrated a good feasibility and safety of the combination DAC + ATRA in the clinical setting [7]. Furthermore, significantly improved survival across risk groups and higher objective response rates were observed for the combination of DAC + ATRA compared with the no-ATRA groups, without added toxicity [2]. The addition of ATRA is most likely impeding the development of secondary resistance to HMAs, possibly through reactivation of ATRA signaling**.** [2]**, **[8] The currently recruiting DECIDER-2 phase III trial has the objective to compare the efficacy of ATRA versus placebo as add-on to DAC + VEN (DECIDER-2, AMLSG 32–21).

Regarding clonal evolution, apart from the initial del(5q), no further aberrations were detected by FISH at the time of transformation to AML; however, 2.7 years later a new clone, which harbored a trisomy 8, emerged. One year before the patient deceased, a new mutation in *ETV6* (p.Y104Pfs*24) was detected. These findings led us to speculate that the combination of DAC + ATRA decelerated the acquisition of new mutations, i.e., “stabilized” the leukemic clone. At time of fully established secondary HMA resistance (5.3 years from AML diagnosis), a *KRAS* mutation had been newly acquired (see below). The acquisition of mutations in *KRAS* in a subset of patients at secondary resistance to HMA has been described previously [9]. Whether this mutation solely, or to what extent the remaining changes in the mutational profile contributed to the secondary resistance is unknown.

In addition to the aforementioned analyses, WES was conducted on blood samples of the patient collected at the time of diagnosis and shortly before she deceased. In total, the AML had gained or lost 1.45 variants/megabase [10]. In order to identify likely pathogenic variants, we focused on cancer genes as defined by OncoKB. The AML had overall gained 4 mutations in cancer genes: *ETV1* (p.R405H, VAF 30.4%), *KRAS* (p.G12A, VAF 27.9%), *PARP1* (c.2277 + 1G > C, VAF 7.5%), and *ETV6* (p.I358M, VAF 5.3%). (Fig. 1C) Interestingly, the *ETV6* mutation was not identical with the previously detected mutation in exon3 at 4.2 years, probably underlining the pressure on the clonal selection of an *ETV6* mutant leukemic cell. *ETV1* and *PARP1* were not covered by the NGS at 4.2 years, while *KRAS* was in wild-type configuration. Thus, we cannot conclude the time of acquisition of *ETV1* and *PARP1* mutation during the disease course, but the AML had acquired a *KRAS* mutation toward resistance. The AML also lost mutations in cancer genes toward the end of treatment in comparison with initiation, e.g., in *IL3* (p.P27S, VAF 9.3%) and *NCOR2* (p.G1830delinsSSGG, VAF 19.6%).

In the decision-making process, we integrated the patient’s priorities and available therapy options, which resulted in more than 5 years OS, with a total of 43 days spent overnight in the hospital and 231 days of outpatient visits for therapy administration, transfusions, or ambulatory check-ups. This case is an example for successfully minimizing the treatment-associated time burden (“time toxicity”) resulting from hospital stays, which is a relevant factor defining quality-of-life for patients in a palliative setting [11]. Of note, this patient’s marked clinical benefit derived from the DAC + ATRA combination treatment (arm C of the DECIDER trial) over years is reflective of the two-year survival probability of all patients on this treatment arm of 25%, which compares favorably to the two-year survival rate of patients in arm A (decitabine-only, < 5%).

In summary, we report on a patient with several high-risk features (secondary AML, adverse genetics, older age) and an above-average survival treated with the combination therapy of HMA + ATRA, continuously administered over 52 cycles. The rate of side effects was kept to a minimum, and quality of life was maintained until weeks before the patient deceased. This case is important as older patients with AML—even in the era of HMA combination therapies—still have a dismal prognosis if allografting is not an option, so the stabilization of their quality of life is one of the central treatment goals.

### Supplementary Information

**Additional file 1.** **Supplemental Table.** List of studies and case series on AML patients receiving HMA therapy.

**Additional file 2.** Supplemental methods.

## Data Availability

The data that support the findings of this study are available on request from the corresponding author, [M.L.].
