# Supplementary material for: Long-term decitabine/retinoic acid maintenance treatment in an elderly sAML patient with high-risk genetics
Source: Clin Epigenetics. 2023 Nov 28;15:185. doi: 10.1186/s13148-023-01596-5 (PMC10683313; doi:10.1186/s13148-023-01596-5)
Supplement: Supplementary file 1 — Additional file 1. Supplemental Table. List of studies and case series on AML patients receiving HMA therapy. [file 13148_2023_1596_MOESM1_ESM.pptx]

## Slide 1
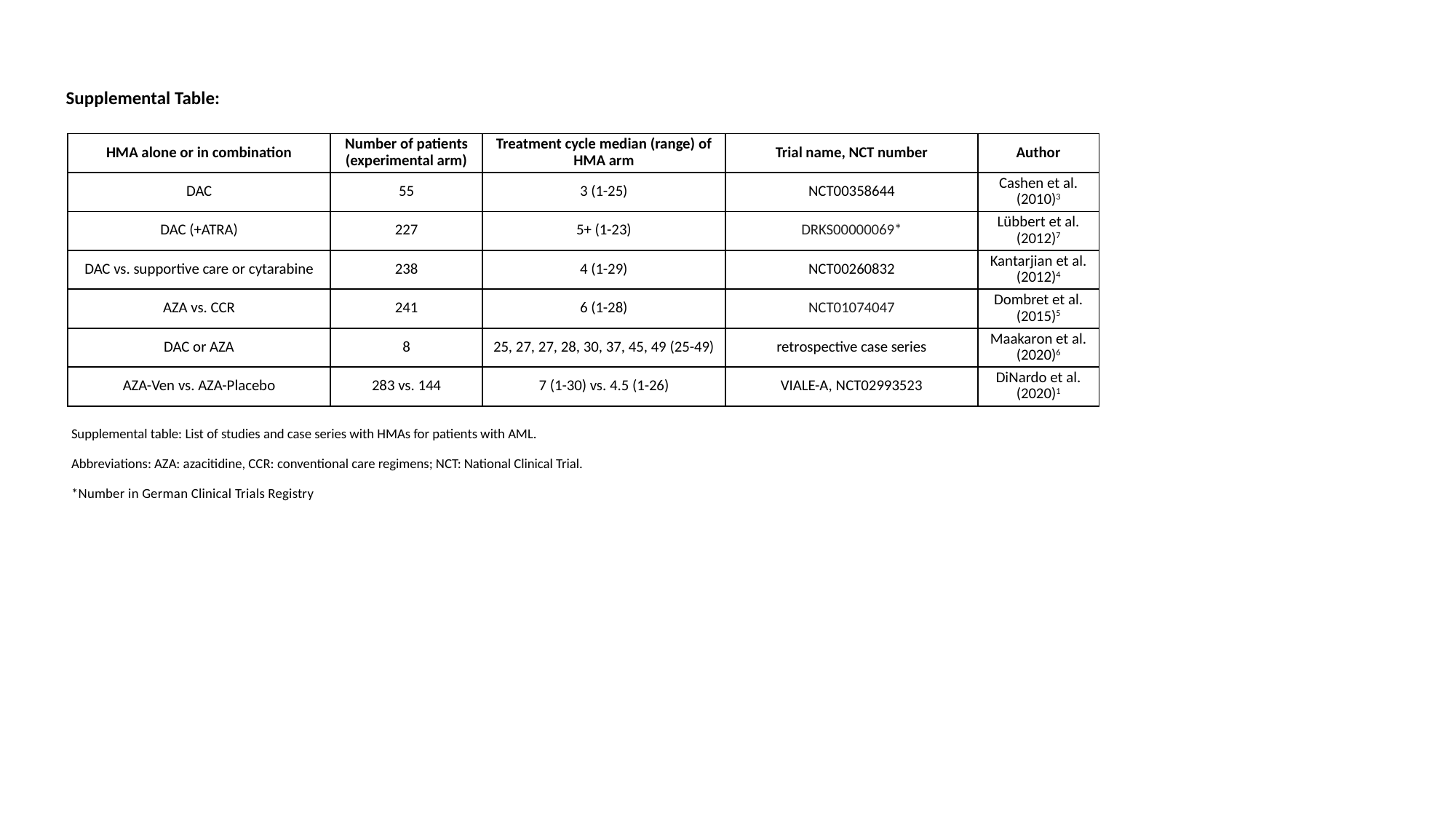

Supplemental Table:
| HMA alone or in combination | Number of patients (experimental arm) | Treatment cycle median (range) of HMA arm | Trial name, NCT number | Author |
| --- | --- | --- | --- | --- |
| DAC | 55 | 3 (1-25) | NCT00358644 | Cashen et al. (2010)3 |
| DAC (+ATRA) | 227 | 5+ (1-23) | DRKS00000069\* | Lübbert et al. (2012)7 |
| DAC vs. supportive care or cytarabine | 238 | 4 (1-29) | NCT00260832 | Kantarjian et al. (2012)4 |
| AZA vs. CCR | 241 | 6 (1-28) | NCT01074047 | Dombret et al. (2015)5 |
| DAC or AZA | 8 | 25, 27, 27, 28, 30, 37, 45, 49 (25-49) | retrospective case series | Maakaron et al. (2020)6 |
| AZA-Ven vs. AZA-Placebo | 283 vs. 144 | 7 (1-30) vs. 4.5 (1-26) | VIALE-A, NCT02993523 | DiNardo et al. (2020)1 |
Supplemental table: List of studies and case series with HMAs for patients with AML.
Abbreviations: AZA: azacitidine, CCR: conventional care regimens; NCT: National Clinical Trial.
*Number in German Clinical Trials Registry
