# Supplementary material for: Long-term decitabine/retinoic acid maintenance treatment in an elderly sAML patient with high-risk genetics
Source: Clin Epigenetics. 2023 Nov 28;15:185. doi: 10.1186/s13148-023-01596-5 (PMC10683313; doi:10.1186/s13148-023-01596-5)
Supplement: Supplementary file 2 — Additional file 2. Supplemental methods. [file 13148_2023_1596_MOESM2_ESM.docx]

**Supplemental Methods:**

Fluorescence *in situ* hybridization (FISH): For FISH analysis the slides were pretreated and hybridized with fluorescence labeled DNA probes: XL 5q31/5q33, XCE 8, XL del (7)(q22q31), XL TP53/NF1, XL TET2, XL MLL, XL MECOM 3q26, XL ETV6, XL FGFR1 (according to the manufacturer’s protocol MetaSystems probes, Germany). Digital images of interphase spreads embedded in Vectashield mounting medium (Vector Laboratories, Burlingame, CA) were recorded with a Sensys digital camera (Photometric,Tucson, AZ) on an Axioplan I fluorescence microscope (Zeiss, Jena, Germany) with Plan-Apochromat 63/40 or 100_/1.30 objectives (room temperature) using the Vysis workstation QUIPS (Vysis, Downers Grove, IL).

Targeted new generation sequencing (NGS): The Illumina TruSight Myeloid panel was used for targeted resequencing and processed as described by the manufacturer (Illumina Inc., San Diego, CA). FASTQ-files were further analysed with the SeqNext software (JSI Medical Systems, Ettenheim, Germany). The Quality Score threshold was set to 30 and the ignore reads threshold to 40%. We used a significance threshold of 3% for the detection of mutations, with a minimum coverage of 200 reads and 5 reads per variant.

Whole exome sequencing (WES): WES was performed by the DKFZ Heidelberg.
